# Children's right to play in times of war

**DOI:** 10.1111/bioe.13232

**Published:** 2023-11-08

**Authors:** Aleksandra Glos

**Affiliations:** ^1^ Center for Bioethics, Faculty of Law, Falculty of Medicine Pontifical Catholic University of Chile Santiago Chile

**Keywords:** child health, children's right to play, children's rights, play, posttraumatic play, war

## Abstract

This paper discusses children's right to play and its bioethical importance for children affected by war. Against the background of the current military conflicts, it analyses physical, psychological, and institutional factors that limit children's right to play in a situation involving armed conflict. Considering that the lack of institutional support of play for children affected by war constitutes a failure to fulfil our societal and political obligation under Article 31 of the United Nations Convention on the Rights of the Child, this paper analyses the understanding of play adopted in this legal instrument. In line with the distinctions made in the Convention, it discusses the importance of play for children's life and survival, for their health and well‐being, as well as for the recovery of trauma experienced by children affected by armed conflict. It thereby aims to contribute to the recognition and protection of this right on the grounds of bioethics and healthcare.

## 1 INTRODUCTION

Children have the right to play. This right is considered a basic human good^1^ and a basic human capability,^2^ without which human life loses its dignity and joy. Arguing for the critical importance of play for children's development, Martha Nussbaum explained: ‘Inability to play or laugh is taken, correctly, as a sign of deep disturbance in an individual child; if it proves permanent, we will doubt whether the child is capable of leading a fully human life’.^3^ The right to play is also a positive right, recognized by the international community and written into the UN Convention on the Right of the Child (UNCRC) of 1989. Its article 31 reads: 4

1. State Parties recognize the right of the child to rest and leisure, to engage in play and recreational activities appropriate to the age of the child and to participate freely in cultural life and the arts.
2. State Parties shall respect and promote the right of the child to participate fully in cultural and artistic life and shall encourage the provision of appropriate and equal opportunities for cultural, artistic, recreational and leisure activity.

However, despite the exceptionally wide, almost universal, ratification of this Convention,^5^ children's right to play is far from being universally recognized, protected and fulfilled. On the contrary, it is considered the most undervalued, neglected 6 or even forgotten right.^7^ In light of the growing understanding of the intrinsic value of play for the dignity of children, as well as its instrumental importance for their development and health, this negligence is a reason for deep concern.

One of the circumstances in which children's play becomes most precarious is during armed conflict. Wars and other forms of political violence curtail physical opportunities to play and exert a psychological toll on children that further limits their capability to do so. The increasing amount of empirical research provides consistent evidence that children exposed to political violence are at risk of suffering from posttraumatic stress disorder (PTSD), depression, anxiety, insomnia, emotional problems and psychosomatic symptoms.^8^ These factors inevitably impede, modify and disturb children's play. Consequences of this disturbance or even absence of play are detrimental. Play is a children's mode of being in the world. As van Gils aptly put it, the right to play is the right to be a child.^9^ By losing this right, children lose an opportunity to develop in a healthy way and thereby also diminish their chances for future physical, mental and social well‐being. However, despite the alarming increase of political violence in recent years, the discussion on the effective ways of respecting and fulfilling children's right to play in situations of armed conflict is largely missing.

This paper draws attention to this neglected problem. Against the background of the current military conflicts, it (1) discusses physical, psychological and institutional factors that limit children's right to play in a situation of armed conflict. This problem refers to all children affected by armed conflicts, including those in actual war zones, as well as children who have fled their war‐torn countries. Although their experiences may differ in duration and gravity, both groups of children encounter substantial physical and psychological barriers to exercising their right to play. However, in discussing our legal obligations to protect children's right to play, the paper will focus on the situation of displaced children who have arrived on the territory of safe countries. Under article 22 UNCRC,^10^ the receiving countries not only have a direct obligation to ensure that refugee and asylum‐seeking children have equal opportunities to enjoy the rights provided for in article 31 as children from the host country,^11^ but they become ‘primary duty bearers to fulfil these rights for the children entering their country’.^12^ Insofar as art. 4 obliges State Parties to undertake concentrated legal, administrative and other measures to implement children's rights, insufficient institutional support of play for displaced children can be considered a failure to fulfil our legal obligation under UNCRC. It is worth mentioning that art. 3 of the UNCRC, which introduces the principle of the best interest of the child and serves as a ‘gateway to children's rights’,^13^ also emphasizes State Parties' active duty to take all appropriate legislative and administrative measures to ensure the child such protection and care as is necessary for his or her well‐being (paragraph 2). The term ‘well‐being’ marks an important shift in our understanding of children's place in society: the shift from protecting children's welfare to advocacy for their rights and well‐being; moving from monitoring a ‘minimum’ necessary for children's survival to a focus on the quality of their lives; and the shift from only objective measurements to those that include children's subjective perspectives.^14^ The concept of child well‐being does not have an official legal definition, and yet, it is usually understood as a positive state (not only the absence of problem or pathology)^15^ that integrates different dimensions of a child's life: their health and safety, cognitive, emotional and social development, as well as their relationships with their family, peers and the wider community.^16^ The holistic nature of children's well‐being obliges State Parties to adopt a comprehensive, multidimensional approach and support activities that nurture children's overall development, such as play.

To make the scope and content of these obligations clear, in the second step, the paper (2) analyses the understanding of play adopted in the Convention, with a special emphasis on the relevance of the right to play for children's health and well‐being. In particular, it reads the art. 31 in conjunction with (a) art. 6 (life, survival and development), (b) art. 24 (highest attainable standard of health) and finally, (c) art. 39 (recovery and social reintegration of children victims of armed conflicts). Following this legal analysis, the paper then (3) turns to the empirical evidence for the importance of play for, respectively, (a) children's life and survival, for (b) their health and well‐being, and (c) for the trauma recovery of children affected by armed conflict. In doing so, it aims to contribute to the greater recognition and protection of this right on the grounds of bioethics and healthcare.

## 2 PLAY UNDER ATTACK

### 2.1 Physical limitations

Play is one of the most immediate victims of war. Even before the first casualties appear, children are rushed to safety, which curtails their opportunities to play outdoors, meet with peers and engage in any unattended, carefree activity of their own.^17^ In contemporary armed conflicts, unlike a century ago, over 90% of victims are civilians, the majority of whom are women and children.^18^ The ongoing war in Ukraine is a good example: children are deliberately targeted (as in the first day's attack on the Kiev maternity hospital and orphanage), or exterminated alongside their parents (as in the brutal Bucha massacre) or forced to live in inhuman conditions (like spending 65 days in one of the Mariupol's bunkers). It is worth noting that, in the latter case, as one Ukrainian mother recalls, hungry and traumatized children did not cease to play, but their games were desperately circulating around two subjects: food and war.^19^ These deplorable conditions are usually followed by a perilous journey to safety, which further limits children's opportunities to play. Also, the condition of being a refugee or asylum seeker often implies a loss of play,^20^ for refugee camps can be dangerous places, exposing children to the risk of violence or discrimination. Integration into a new country does not come without difficulties and may entail prolonged periods of isolation and distress.^21^ Moreover, refugee families often live in cramped living conditions that lack safe spaces to play.^22^

### 2.2 Psychological toll on play

Even more burdensome than the physical limitations is the psychological toll that war exerts on children's play. Children often fall victim to war crimes: United Nations verified 27,180 cases of grave violations against children in 2023, including killing (2985), maiming (5655), followed by the recruitment and use of 7622 children by armed forces in conflict and 3931 incidents of denial of humanitarian access, and many brutal instances of rape, sexual violence and abduction.^23^ Many children are also traumatized by having witnessed crimes committed against their loved ones. Not less disturbing is the atmosphere of insecurity and chaos, which war, with its constant threat to life, displacement of populations, social and infrastructural breakdown, inevitably produces.^24^ Living under conditions of ‘mass trauma’ may upset children's basic need for safety^25^ and imprint in their minds a deep conviction that the world is not a safe place, and people are not to be trusted. The secondary effects of war are also distressing: for example, most of the youngest Ukrainian refugees are separated from their fathers who stayed in the country to defend it,^26^ and in some cases, even when mothers are present, they can fail to take proper care of the child due to their own emotional suffering or bereavement. This has, in fact, been recognized as one of the major factors of developing early and chronic psychopathology.^27^

The above descriptions are only a few of the brutal ways by which war affects its youngest victims, but they leave no doubt that their psychological effect on children is severe. In the wake of traumatizing war events, children suffer from posttraumatic stress disorder, depression and anxiety disorders, sleep problems, chronic stress, aggressive behaviours, school refusal, loss of appetite and other somatic symptoms.^28^ The gravity of these symptoms varies from being discomforting, to disturbing or seriously disabling a child's functioning.^29^ These mental health conditions also invariably affect children's play. The literature refers to the changes in play of the children exposed to traumatic events as posttraumatic play (PTP).^30^ One of its most common symptoms is re‐experiencing the tragic events through play. Contrary to what is popularly assumed, children do not forget traumatizing events, but are often tormented by flashbacks, dreams or memories (‘It is like having a camera in my head’,^31^ as a Bosnian child explained). Clinical literature distinguishes two types: positive (or dynamic) and negative (toxic) play.^32^ The positive type is when children re‐enact the frightening events in their play but are able to modify its negative components and gain control over the experience. Negative play, however, re‐enacts the traumatizing images and feelings but fails to resolve them and relieve anxiety. Other typical consequences of trauma among children are a grim and despairing quality to their play, increased aggressiveness in their play, fantasies around revenge, reduced reactions and desensitization to the environment or, contrarily, being constantly alert to possible danger.^33^ In the most severe cases, children may suffer from a temporary inability to play or inability to play symbolically. As Launders explains: ‘Trauma may have interrupted developmental processes to the extent that symbolic capacities were not generated. Early relationships may have been disrupted, preventing the child from using transitional objects and other toys as symbols for significant people and experiences’.^34^ On the grounds of Winnicott's^35^ theory of play evoked in this quote, the capacity to engage in symbolic play is formed in early childhood when children use the first transitional objects (e.g., a blanket, a piece of wool, a teddy bear, being a symbol of the union of a baby with a mother) to comfort themselves when mom is absent. In the course of children's development, symbolic or pretend play progresses to more advanced stages and children begin to use objects to symbolize other objects (e.g., using a banana as a cell phone) or engage in complex pretend behaviours (e.g., playing ‘mom’ or ‘dad’ in an imagined, home‐like scenario).^36^ Symbolic play has been argued to be a necessary component in the cognitive and emotional development of children.^37^ In Winnicott's theory, the usage of symbolic objects conditions children further entering into a potential space,^38^ an intermediate area located between imagination and reality,^39^ where they can both experience his or her own creative powers (invent their own roles, imaginative scenarios, or, in the postwar context, replay their traumatic memories into a soothing, coherent narrative) as well as enjoy the products of others' creativity in a cultural experience. In that sense, play deprivation may have a critical effect on the whole life of a child in its most meaningful aspects.

### 2.3 Institutional factors limiting children's opportunities to play

Children's play is limited not only by physical and psychological circumstances of war but also by the inadequate protection and provision offered by political and social institutions. To understand the scope of this problem, it suffices to take a quick look at the state of implementation of article 31 of UNCRC by State Parties. In 2013, the Committee on the Rights of the Child issued General Comment on art. 31,^40^ which raised awareness regarding the lack of recognition of the significance of play in the lives of children, lack of appropriate provisions and weak or non‐existent protective legislation. The Committee also expressed its concern about the unequal enjoyment of this right by children in situations of conflict, as well as child victims of humanitarian and natural disasters. The recent comparative study^41^ on the compliance with art. 31, based on the reports provided by State Parties, proves that, despite these efforts, children's right to play can be still described as the ‘least known, least recognized and least understood’^42^ right of childhood. According to the study, the majority (69.23%) of countries that delivered reports do not even mention children's right to play once. From those few countries that do mention it, most report only scattered initiatives introduced to support play in the educational and social spheres. In the words of the authors: ‘There are virtually no comments on laws and regulations that encourage, finance or regulate the child's right to play’.^43^ Only two countries adopt coordinated policies (Ireland and United Kingdom), which, however, pay limited attention to the play of child refugees and asylum seekers. Moreover, the same study^44^ reports a significant lack of monitoring play interventions and almost a complete absence of indicators to measure free‐time activity of children, which makes it practically impossible to evaluate and improve the enforcement of the right to play in a systematic manner.

In the absence of coordinated, national strategies to promote children's play, insufficient consideration for the play of the displaced refugee and asylum‐seeking children is no surprise. This does not mean that play is unprovided^45^—the primary mechanism to promote children's psychosocial well‐being in emergencies are Children Friendly Spaces (CFS), which, since the Kosovo conflict in 1999, has been successfully adopted by UNICEF and other non‐governmental organizations. However, the systematic review of outcomes of these interventions (based on existing literature describing CFSs and documented reports delivered by NGOs active in the use of CFSs in emergency contexts) demonstrated significant shortcomings in this case as well.^46^ Even if the outcomes of these interventions are reported to be positive, the study shows that the evaluation of these outcomes lacks standardized and rigorous criteria of measurement, scientific research on the subject is limited and children are not consulted regarding the design, monitoring and evaluation of the process. It is worth noting that even if the play provision is an important part of CFSs, the study does not mention this particular intervention.

Concurrently, the recent observational studies devoted to the specific topic of play in refugee camps in Greece give reasons for concern. It is reported that CFS prioritized structured, adult‐led activities and psychosocial support over free, child‐directed and initiated play.^47^ Programmes are tightly scheduled and colonized by adult psychological or educational interventions, not leaving enough space for children's liberty to invent their own play. According to Ardelean,^48^ reasons for this neglect of free play lie in the lack of holistic understanding of play observed in CFS and respective lack of training for staff and volunteers working with children at play. This gap in theory and practice results in difficulties with accommodating violent games of refugee children. As Landers notes, ‘Children who come from dangerous environments play in dangerous environments’.^49^ This fact may, however, present a challenge, especially for the untrained staff, or for those trained in the ‘zero tolerance’ approach to children's violent play. The question posed by Waniganayake poignantly expresses this challenge: ‘how would you react when observing refugee pre‐schoolers quietly and seriously pretending to slit each other's throats?’^50^ The author reports that in the described case, the staff, not without difficulties, abandoned the policy that prohibited violent play, managed to accommodate the anger and gradually directed them to nonviolent alternatives. However, other authors account for a more hesitant^51^ or even prohibitive^52^ approach to the problem of violent play, which, even if discussed in the wider literature, did not receive enough scholarly attention in the specific context of war‐related posttraumatic play, where it is most ubiquitous. As Ardelan rightly concludes, there is an urgent need to adopt a research‐informed play policy that would include minimum international standards for play provision in refugee camps and could guide caregivers through specific challenges that arise in this precarious context.^53^

### 2.4 Summary

War limits children's access to play. Eight‐year‐old Sasha from Ukraine described a typical problem that may arise in a war zone: ‘While at the playground I saw three missiles flying above me’.^54^ Clearly, bombarded playgrounds, bunkers and besieged cities do not offer safe places to play. Also, refugee and displaced children, after fleeing to safety, may experience barriers to play resulting from their difficulties to adapt to a new country, not knowing its language and not having anywhere to play. (‘There are no trees to play under and no playground to go to’,^55^ as a 10‐year‐old girl in a transit centre for internally displaced persons in Sri Lanka put it). Apart from physical limitations, tragic war events have a devastating influence on children's mental health. Children exposed to political violence are at high risk of posttraumatic stress disorder, depression, anxiety and other mental health issues, with potential long‐term effects and implications. The mass trauma of war also affects children's play, tormenting their games with turbulent memories and emotions or, in the most serious cases, depriving them of the ability to play. Unfortunately, children's right to play, despite being one of the most basic rights of childhood, tends to be neglected in state policies and international strategies. Considering that—under article 22 of UNCRC—the countries receiving refugees have the primary duty to protect children's right to play, the next section will explore the meaning of play adopted in this Convention and analyse our legal obligation stemming from it.

## 3 CHILDREN'S RIGHT TO PLAY IN UNCRC

Play is a notoriously ambiguous concept.^56^ We seem to understand play intuitively, particularly while engaging in it (with children thus being the best experts in the matter), but we can easily flounder when we try to define it. In light of this ambiguity, what follows is a discussion of what has until now been the most operationalizable understanding of play—the one adopted in UNCRC, interpreted, however, against the wider theoretical background. After discussing the concept of play adopted in UNCRC, this section will focus on analysing our legal obligations to provide play resulting from this Convention.

### 3.1 Intrinsic value of play

Contemporary definitions of play and its role in human societies oscillate between two general approaches: the first described as cultural or intrinsic, and the second called developmental or instrumental.^57^ The first understands play as a foundation of culture and underlines its intrinsic value for the pleasure and dignity of a human person. As Huizinga^58^ famously argued, play is the constitutive element of such cultural expressions as games, sports, theatre, dance, literature and other arts, language and its normativity (a good example is Wittgenstein's concept of the language game, whereby the meaning of words is not set in stone, but emerges from the rules of the game played by language users^59^), philosophy, law and religious rituals (e.g., Platonic *theologia ludens* 60 or Guardini's understanding of liturgy as divine play^61^). Consider the ubiquitous character of children's pretend play, where children with all seriousness behave ‘as if’ they were astronauts in a spaceship, or princesses in a castle—this ‘as if’ attitude, which can be interpreted as the *differencia specifica* of play,^62^ develops in adult life into a complex intellectual operation of inventing cultural artefacts of creative fictions in science.^63^

In opening the space for creative potentiality, play also becomes a springboard for children's freedom. While drawing from the cultural heritage of their communities, they create their own worlds, construct their own spaces made of wooden sticks, sand or Legos and are able to write their laws, roles and stories. In this way, they build and exercise their own agency. Play is the exercise of freedom also in the sense that it has to be voluntarily chosen and intrinsically motivated; if it is imposed against a child's will, it ceases to be play. The value of play, despite its countless developmental benefits, does not lie in any external goal, but in the intrinsic thrill of it (‘The joy of play is the ecstatic feeling of liberty’,^64^ as one of the most ardent defenders of children's freedom to play, Peter Gray, put it.) Children play because it is fun, and in doing so, move beyond purely instrumental or utilitarian logic. In that sense, allowing children to play, and, in particular, to play freely, is a way to express and transmit respect to their human dignity.

### 3.2 Instrumental approach to play and its limits

The second, developmental or instrumental approach, in contrast, emphasizes the external benefits of play in the development of cognitive skills, social capabilities and the well‐being of children. It treats play as an instrument to achieve these developmental ends (and not as an end in itself). This approach is well grounded in empirical research in the field of child psychology, as well as the cognitive and evolutionary sciences.^65^ Best documented is the value of play for cognitive and educational development: for at least few decades,^66^ play has been considered as the means though which children learn.^67^ This is the reason why play has been harnessed to the educative framework, why today's schools are increasingly introducing various form of play‐based learning.^68^ The instrumental use of play in the school setting is also reflected in state policies. As Payà and Bantulà demonstrate, several State Parties report having undertaken various initiatives to implement children's right to play in the area of education.^69^

However, the instrumental use of play has its limits. The example from education well illustrates this problem. The point has been raised that play‐based learning initiatives usually adopt an adult perspective on play, instead of consulting children on the matter.^70^ To understand children's perception of play, Howard, Jenvey and Hill^71^ conducted a study where they showed children photographs of activities in a classroom setting, asking them whether they play or not play. The study revealed that children usually considered an activity to be play where a teacher was absent; if the teacher was present, activity was judged as non‐play (even if children in the photograph seemed to enjoy it). Importantly, it turned out that only play‐like scenarios release the full developmental potential of play. In their most instructive study, Howard and McInnes^72^ compared two groups of children. Both were engaged in the same activity: jigsaw puzzle and bead threading. However, one group performed this activity in the ‘like play’ mode (an adult was nearby, activity occurred on the floor and the child had a choice regarding participation) and the second in the ‘not like play’ mode (an adult directly present, activity occurred at a table and child was asked to take part). It was found that when an activity was perceived as play, children's emotional well‐being increased and more cognitive benefits were gained (children were more attentive, focused on activity and motivated and most importantly—smiled more). This constitutes an important lesson to bear in mind when including play in developmental programmes, be it in educational or health‐related contexts to be discussed below. The paradox of play is that its developmental benefit hinges upon its retaining intrinsic value for children, them regarding it as play and keeping, as Bogatić aptly put it, a ‘sense of ownership of play’.^73^ Speaking in terms of rights, children's right to play should not be violated through the instrumentalization and pedagogization of play. This does not mean that the play cannot be provided with developmental gain in mind, but it constitutes an important caveat to it: in harnessing play to any developmental schemes, we have to listen to children's own preferences and choices in play.

### 3.3 Definition of play in UNCRC

Let us now see how the interplay of both approaches is reflected in UNCRC. The understanding of play adopted in the Convention fits into the cultural approach to this phenomenon. Article 31 of the Convention separates play from duties and necessities of daily life, and places it in the context of recreation, leisure and cultural and artistic activities, also emphasizing children's freedom in taking part in these. The second paragraph additionally emphasizes children's participation rights that should be promoted by State Parties. Such a construction of the article, whereby play is integrated into the sphere of cultural and artistic life, indicates that the Committee on the Rights of the Child conceptualizes play in frames of the first of the approaches described above, namely, cultural or intrinsic. As General Comment to the art. 31 emphasizes, each element of the article is ‘mutually linked and reinforcing’, in particular: ‘play and recreational opportunities provide the conditions for creativity’, while ‘immersion in cultural life enriches playful interactions’.^74^ According to Van Gils, this holistic reading suggests that ‘Play and recreation and cultural and artistic activities belong to the same category: to culture in the broad sense of the word’.^75^ Belonging of play within a cultural framework is also visible in the definition of play developed in General Comment. While defining play, General Comment emphasizes such elements as its intrinsic value, voluntariness and the agency of children. Children's play is defined as ‘any behaviour, activity or process initiated, controlled and structured by children themselves’^76^ that is ‘driven by intrinsic motivation and undertaken for its own sake, rather than as a means to an end’.^77^ Play, as the Committee further states, ‘involves the exercise of autonomy’ and its ‘key characteristics are fun, uncertainty, challenge, flexibility and non‐productivity’.^78^

### 3.4 Play and children's right to be heard

It is a widely acknowledged principle of human rights that they are interrelated, interdependent and indivisible.^79^ General Comment to art 31 is the best example of such interdependent reading of this provision. It states that children's right to play should be read not only in the perspective of the whole art. 31 but also in conjunction with other articles of the Convention. One of the main principles in light of which the right to play should be interpreted is children's right to be heard (art. 12). The right to be heard is the core children's right expressing respect for their agency. As Archard aptly put it, ‘a child's right to be heard on matters affecting its own interests is a substitute for the liberty right to make one's own choices’.^80^ In other words, children might not be able to make choices that adults are permitted to make, but they have a right to express their voice on matters that affect them. Moreover, the value of children's voice is not purely consultative^81^; the voice of children has normative power: it should be listened to, given ‘due weight’ in consideration and, whenever it is also in accordance with the best interest of a child (art. 3), acted upon. In reference to art. 31, this means that children are entitled to exercise choice and autonomy in their play. Furthermore, the Committee emphasizes that the voice of children should be listened to not only in their individual play decisions (such as, for example, what kind of game children want to play in a refugee camp) but should also influence the development of legislation, policies, strategies and design of play programmes, and other relevant documents to promote children's right to play.^82^ The obligation to listen to children when organizing their play activities is further strengthened by the unique nature of this right. As Alen et al. notice, children's right to play is ‘a new type of right (…);’ ‘a right only acknowledged under international law for persons below 18. No other human rights instruments do recognize this right, though some soft law instruments do promote the right to play’.^83^ In other words, children's right to play belongs to children; it is the right that allows a child to be a child, and as such, it requires adults to show particular respect towards children's autonomy in the exercise of this right.

### 3.5 Instrumental value of play in UNCRC

While emphasizing the intrinsic value of play, the Committee on the Rights of the Child does not neglect the developmental (instrumental) functions of play. On the contrary, it proposes a well‐balanced equilibrium between the understanding of play as being foundational to the ‘pleasure of childhood’ and as being ‘an essential component of physical, social, cognitive, emotional and spiritual development’^84^ of a child. The Committee emphasizes two instrumental uses of play: first, its importance for children's education and physical, social, cognitive and emotional development, and, second, for the health and well‐being of children. As it was argued, in the literature, the importance of play for the educational formation of the child is well documented. It is also the one that is implemented in many state policies. Much less attention, however, has been paid to the relevance of play in the health care sector. This is surprising, if we take a closer look at the many ways in which the Committee on the Rights of the Child discusses the relationship between children's right to play and their right to health.

### 3.6 Play and children's right to health

The Committee of the Rights of the Child emphasizes that ‘play and recreation are essential to the health and well‐being of children’.^85^ General Comment makes three references to children's health that are particularly important for the well‐being of children affected by political violence and make clear the contribution of play to the well‐being of children. First is the reading of children's right to play in light of their right to life and the maximum possible extent of survival and development (art. 6), which is one of the fundamental principles of the Convention.^86^ By placing life, survival and development next to each other, this principle seems to lead the Committee to adopt a holistic approach to human well‐being and sees as equally important—and mutually reinforcing—the obligations to support the biological survival of children and to create opportunities for them to thrive.^87^ As Collins and Wright^88^ rightly observe, reading art. 31 in view of the principle of survival and optimum development emphasizes the importance of the right to play in the international humanitarian sector, which, by definition, deals with situations where the survival and healthy development of children are endangered. In this context, it is also crucial to respect children's autonomy in play. While interpreting the right to play in light of art. 6, Lester and Russell argued that play contributes to the survival of children because it gives them an instrument of ‘self‐protection’.^89^ In play, they explain, children create their own well‐being and use their own inner resources to develop the resilience necessary to be able to live through adverse situations.

Second, the Committee underlines the importance of article 31 for the child's enjoyment of the highest attainable standard of health recognized in article 24, which, in paragraph f, also encompasses the dimension of preventive health.^90^ General Comment reiterates the importance of play for the holistically conceived health of children (it reads: ‘(…) the realization of the rights provided for in article 31 contributes to the health, well‐being and development of children’^91^). It also draws attention to the rehabilitative potential of play, stating that children's ability to enjoy their right to play when they are ill or hospitalized has ‘an important role in facilitating their recovery’.^92^ The importance of children's right to play for their health has also been emphasized in jurisprudence.^93^ As Mullen^94^ argued, a failure to ensure the human right to play leaves entire populations at a heightened risk of obesity, non‐communicable diseases and various threats to mental health. Accordingly, he proposed to include play programmes in public health strategies, justifying that only population‐wide instruments of public health approach can ‘ensure the universal enjoyment of play as a human right’.^95^

Finally, the Committee points to the therapeutic value of play for the mental health of children affected by war and conflict, as emphasized both in legal doctrine and in empirical studies (to be discussed in the next chapter). General Comment extensively discusses the right to play in the context of the right to physical and psychological recovery of a child victim of armed conflicts, expressed in article 39. Referring to this article, it reads: ‘Children's experiences, including those which are painful and damaging, can be communicated through play or artistic expression. Opportunities to realize the rights under article 31 can provide a valuable means through which children can externalize traumatic or difficult life experiences in order to make sense of their past and better cope with their future’.^96^ Here again, the Committee emphasizes the agency of children by calling play in this therapeutic context ‘a natural, self‐guided, self‐healing process’.^97^ While discussing the value of the right to play for children's mental health, Shackel^98^ underlined its meaning‐making role. Play is where children can process what happened to them, position themselves with respect to the world and attach personal meaning to their experiences. Many scholars^99^ point out the therapeutic value of joy and the pleasure of play. Feelings of joy and happiness help children to achieve a sense of normalcy again after traumatic experience, and gives them resources to mitigate the effects of stress as well as overcome adversity. In addition to this potential of play to be a self‐guided and self‐healing process, the Committee also sees it as a possible instrument in the hands of professional therapists. ‘Play enables children to communicate their experience’,^100^ and that, as Shackel explains, makes it a ‘therapeutic medium’^101^ that can reveal a child's mental life—with all its conflicts, tensions, defences and potential solutions.

### 3.7 Conclusions

The above analysis amounts to the conclusion that State Parties' obligation to respect, protect and fulfil children's right to play also extends to the healthcare sector. In its reading of art. 31 in conjunction with art. 24, the Committee of the Rights of the Child emphasizes the importance of play for the health of the entire population of children, while the context of art. 39 adds a specific obligation to realize the right to play for child victims of war and other forms of adversities. However, despite these indications, State Parties' initiatives in this domain remain scarce. In their study on countries' implementation of art. 31, Payà and Bantulà report that the existing initiatives to realize this right almost entirely concentrate in two areas—in the social sphere (e.g., urban planning for play, play in public spaces) and, to a lesser extent, in education.^102^ The absence of reference to the healthcare sector indicates that the provision of play is overlooked in this vital area of state policies. If play is recognized in the health sector, it is mainly in a hospital setting. The provision of play to hospitalized children has been made a WHO standard of children's rights in hospital.^103^ Moreover, article 7 of the Charter for Children in Hospital was adopted by the European Association for Children in Hospital in 1998 to ensure that children ‘have full opportunity for play, recreation and education suited to their age and condition and shall be in an environment designed, furnished, staffed and equipped to meet their needs’.^104^ These regulations attest to the healthcare sector's growing recognition of the right to play, and at the same time, point out areas where its importance is still neglected; for outside this specific context of hospitalization, there are almost no interventions reported in the areas such as public health, or other general and mental health policies.

However, in recent times, there has been an increase in scholarly and institutional voices addressing this gap and advocating for systematic inclusion of play in health‐related policies. The first group of studies postulates the provision of play opportunities as a part of public health strategy. Within this framework, Tonkin and Whitaker^105^ most extensively argued that play can contribute to each of the three pillars of public health's engagement in the promotion, prevention and protection of health. For example, it has been demonstrated that play can contribute to the promotion of healthy brain development^106^; it can be a creative and cost‐effective means of preventing obesity and reducing health inequalities^107^; and finally, it can contribute to protecting children from diseases and infections by using play‐based means to educate them in the principles of personal hygiene.^108^ The second group of studies advocates for the inclusion of play in general medical policy and practice. Most famously, in its two recent clinical reports,^109^ the American Association of Paediatrics emphasized the importance of free and child‐centred play for children's healthy child development and urged paediatric health professionals to advocate for this type of play within families, school systems and communities. Interestingly, they also discussed the correlation of contemporary pressure on school achievement at the expense of play to the decline in young people's mental health.^110^ This leads to the third group of studies, discussing children's right to play in the health‐related context. There is a consistent body of evidence attesting to the effectiveness of play and play therapy for recovery from mental health problems, including war‐related trauma.^111^ For example, the recent study coauthored by the Board and Policy Divisions of The European Society for Child and Adolescent Psychiatry (ESCAP),^112^ urging health systems in Europe to meet the increased mental health of children affected by the recent war in Ukraine, recommended providing refugee children with safe places to play as one of the basic means of facilitating their trauma recovery.

The present paper contributes to this emerging bioethical consideration of play by adopting a human rights‐based approach. It argues that children's right to play as defined by UNCRC and interpreted in General Comment gives us a good instrument to justify and set instructions for the recognition and promotion of this in the healthcare sector. To make the legal argumentation presented above more vivid, the next section fleshes out each of the points made here with evidence‐based meaning and experienced‐based illustrations. Referring to the distinctions made by General Comment, it discusses philosophical and empirical arguments to support the claim that realization of children's right to play will contribute to children's survival (as discussed above in conjunction with art. 6), health (art. 24) and recovery from the trauma of armed conflict (art. 39). It also follows the Convention in its attempt to balance instrumental and intrinsic aspects of play, so that the inherent value of play for children—and thus the children's dignity—can be respected when implementing play into their therapeutic framework.

## 4 CHILDREN'S PLAY AS A BIOETHICAL CHALLENGE

The importance of play for a child's survival, health and recovery from traumatic events, recognized by the Committee on the Rights of the Child, clearly demonstrates that providing the opportunity for play for children affected by armed conflict is not only educational but also a bioethical challenge, falling under the obligations of our healthcare systems. The section below will present further, empirical and experience‐based arguments to support this claim.

### 4.1 Play and survival

In Krakow's former Schindler's Factory—now transformed into the Historical Museum commemorating the time of city occupation 1939–1945—one of the video installations presents the story of a life‐saving game.^113^ The story is told by a famous Polish neuroscientist Jerzy Vetulani (1936–2017), who recalls a summer day in 1943, when he, his younger brother Jan, together with their mother Irena, were walking near the Rudawa river. At one point, the mother noticed that Nazi soldiers were guarding the shore of the river and preparing some dangerous operation. Determined that her 7‐ and 4‐year‐old sons not feel any distress in getting home safely, she proposed that they ‘play Indians’, jumping over fences and hiding in backyards, running away from danger. The boys were ecstatic at the suggestion, and eagerly followed their mother in what they thought was tremendous fun, unaware that they were truly running for their lives.

This scene is a prime example of the life‐saving potential of play for children. One of the reasons for the insufficient provision of play may be its assignment solely to the category of ‘thriving’, as opposed to that of ‘surviving’. We tend to understand play as a form of surplus, a luxury, analogous to adults enjoying a card game after a day of hard work.^114^ In times of peace, this understanding results in a growing number of chores, courses and other after‐school activities, not leaving enough time for children's unrestrained and free play. In times of war, when children are losing lives, homes or parents, this view may lead to a dismissal of play in favour of more urgent, and seemingly more serious needs. Play, however, as philosophers such as John Finnis^115^ or Martha Nussbaum^116^ rightly claim, is a basic good, a basic human capability that should be counted among the minimum number of justice‐based entitlements.

Contemporary sciences flesh out this philosophical intuition with compelling empirical evidence. Probably the most convincing argument on the importance of play for survival comes from evolutionary biology. As Burghardt and Graham argue, if play did not have critical adaptive benefits, its high costs in terms of time, energy, risk of injury or predation would lead to selection against it.^117^ However, the contrary is the case. A growing number of ethological studies clearly demonstrate that play is ubiquitous in the animal kingdom: not only do most mammals play but also certain birds, fishes, insects and cephalopods.^118^ Another, nondirect argument for the importance of play for survival comes from observation of consequences of playlessness. Studies on play deprivation among monkeys and rats concurrently suggest its detrimental impact on social competence and stress management. Animals that have been prevented from playing with peers as juveniles are unable to regulate emotions, interact socially with others or to mate successfully.^119^ They also lack what Spinka et al. called ‘training in the unexpected’,^120^ meaning that they are unable to deal with novel, unexpected events without being overly stressed.

In line with these findings, Lester and Russell argue that the adaptive role of play in the human world lies in shaping children's resilience to stressful and novel situations.^121^ One way, among many others, to build this resilience is to train the stress response system. To be fun, play must include some degree of risk and challenge, which generates a situation of temporary, moderate stress. As the authors explain, this experience elevates the level of cortisol and builds new neural connections, which prepares the brain to deal with stressful situations in the future. Thanks to its ‘as if’ frames, play offers a safe ground to develop and test different responses to stressful situations, increasing a child's confidence in their ability to deal with them in the future. Handling stress in playful conditions also links an essentially negative state of being (stress) to a positive affect, which may contribute to seeing future stressful situations as less intimidating.

This adaptive, resilience‐building function of play cannot be overestimated in the case of child refugees struggling with the trauma of war and challenges of dislocation. Responding to the refugee crisis caused by war in Ukraine, Bürgin et al. argued that mental health interventions addressing specific needs of child refugees should be trauma‐informed, that is, grounded in the understanding of the impact that trauma has for the mental health condition of its survivors and responding to it with resilience‐oriented interventions.^122^ Providing safe spaces to play was mentioned as one of the first imperatives of such a resilience‐oriented care offered to traumatized refugee children, considered as a form of ‘psychological first aid’, offered together with such basic services as food, shelter and medicine. Within this framework, play is argued to be one of the means to respond to children's most basic needs, restore their sense of security and bring back some normalcy to their war‐torn lives.^123^ As UNICEF also observed, ‘adding stimulation and play activities in the children's early years to nutrition, health and rehabilitation programmes appeared to speed up recovery among children affected by conflict’.^124^

#### 4.1.1 Summary

In the serious business of supporting children in humanitarian crises such as natural disaster or political conflicts, children's right to play is often belittled or even ignored.^125^ After all, it is the least understood of children's rights, which tends to be easily forgotten even in times of peace. This negligence stems from a misconception of children's play as a privilege or luxury that children must earn. However, as many philosophical and empirical studies demonstrate, play is children's most vital need. It helps them to adapt to new environments and overcome stress in adverse situations. This resilience‐building function of play makes it particularly important in a situation of severe stress and trauma. This is why opportunities to play should be provided to children affected by the trauma of war as a part of a programme responding to their most basic needs. As the opening scenario of play amidst the dangers of World War II metaphorically suggests, play can save a child's life.

### 4.2 Play and health

a. Physical and mental health
  Play has a potent, direct and long‐term impact on children's physical and mental health, as well as on their cognitive and social development. The most immediate impact is its contribution to children's physical health and their senso‐motor skills. Children jump rope, chase each other, climb trees, ride bikes or wrestle with peers, thereby developing their muscles, exercising their respiratory system and improving their overall physical condition.^126^ It is no surprise, therefore, that the American Academy of Paediatrics endorses play as a way not only to strengthen the child's body but also to prevent or reduce obesity with its related health risks.^127^ It is worth mentioning that outdoor playing and other forms of physical activity contribute to the development of the brain and improve children's cognitive performance and emotional well‐being.^128^
  A famous quote by Sutton‐Smith expresses concisely and in stark terms the importance of play for children's mental health: ‘the opposite of play is not work; it is depression’.^129^ The first channel of this ‘anti‐depressive’ function is the positive affect that play fosters. Naturally, the dominant emotion of play is fun. While playing, children enjoy the agility of their bodies, laugh with their friends and jump about excitedly. Through positive affect, play decreases mental distress,^130^ produces patterns of thought that are more flexible, creative, open to the environment^131^ and, as such, leads to better coping, and, on a physical level, to significantly better health outcomes and longer life.^132^ Frederickson explains that positive affect loosens the narrow behavioural grip that a negative affect can have over the body and the mind (e.g., fight or flight reaction to fear) and broadens the repertoire of thought and action, thereby building personal strength and resilience.^133^ As Frijda's classic work on laws of emotions proved, time does not heal wounds, and children do not automatically forget or grow out of trauma.^134^ Traumatic events will retain their power to elicit negative emotions for an indefinite period of time, unless counteracted by repetitive exposure to positive emotional states. This means that to recover from trauma, children need to be given an opportunity to situate themselves—through play—‘in a better state of mind–body–environment interaction’.^135^
  Play, however, is not always fun. Children get bruised falling off bikes, they lose a game while playing with a companion or engage in pretend play oscillating around difficult themes. Paradoxically, these less pleasurable emotions experienced during play can also greatly contribute to the overall emotional well‐being of children. For in play, emotions that would otherwise overwhelm a child are kept in safe ‘as if’ frames. As Sutton Smith explained, play is a ‘parody of emotional vulnerability’,^136^ meaning that certain emotions are evoked without being fully experienced. In this way, play gives a child an opportunity to master primary emotions that are evolutionarily wired into our nervous systems and tend to provoke strong responses (such as fear, anger, disgust, shock, sadness) with more sophisticated emotions and cognitive states such as ‘performance strategy, courage, resilience, imagination, sociability and charisma’.^137^ For example, competitive games mimic the emotion of anger and provide an opportunity to master that anger through strategy and skill.
b. Cognitive and social development
  According to Lester and Russell, it is primarily through the above‐described emotional fine‐tuning that play also contributes to the cognitive development of children.^138^ By preparing them to deal with novel and uncertain situations, play instils in children a readiness to explore new experiences and an openness to new ways of thinking and behaving. Play enhances curiosity,^139^ imagination and creative thinking,^140^ helps children to focus and hold their concentration^141^ and in this way improves their learning performance.^142^ As the primary form of storytelling, play also draws a child into the world of language, logic and literacy.^143^ According to Farné, an important pedagogical circumstance of play is that children are released from adult supervision.^144^ Play—he argues—develops its best educational potential precisely when children escape adults' control and manage to follow their own games based on free decisions and negotiations with peers.
  Interacting with other children in play has a major impact on the development of social capabilities.^145^ Children are learning to communicate, to negotiate, to cooperate, to argue, compromise and to resolve conflicts. In other words, they learn how to play social chess.^146^ Importantly, through play, children enter into the world of norms, learn to follow rules and get a chance to discover the beauty of fair play. Most importantly, through play, children form first attachments and friendships, which, as Sias and Bartoo put it, are a psychological ‘vaccine’ against both physical and mental illness.^147^ In a unique longitudinal study of war‐exposed children across the first decade of life, children's social engagement turned out to be one of the main factors protecting them from developing psychopathology later in life.^148^
c. Play as a social determinant of health

The above‐described benefits of play for the health and well‐being of children are already a good reason for promoting children's right to play in the healthcare sector. Nielsen, however, formulates an additional argument, which is particularly important in the context of socially disadvantaged refugee and asylum‐seeking children.^149^ Play, he argues, is a social determinant of health and, as such, entails an obligation on the part of healthcare systems to promote and facilitate children's play as a way of preventing health inequalities. The basis for this claim is the instrumental importance of play for developing children's social capabilities and cognitive skills. Referring to Wolf and de Shalit's concept of fertile functioning (understood as ways of functioning that, having emerged in one area, are likely to fertilize other areas as well^150^), he claims that play is such a fertile functioning because it enhances children's cognitive development, education and social capabilities. Referring to empirical evidence, he then argues that education, employment and social inclusion are socioeconomic factors that very strongly influence our level of health. It follows that, through enhancing children's social and cognitive capabilities, play also raises their chances for health and well‐being in the future. From there, Nielsen concludes that healthcare systems, which are obliged not only to provide treatment but also, through public health means, to secure equal distribution of social determinants of health, have a specific duty to protect and promote children's opportunities to play.^151^ This argument seems to be particularly relevant in the case of vulnerable populations of refugee and asylum‐seeking children. Their mental trauma and physical exhaustion are usually accompanied by socioeconomic disadvantages (e.g., high degrees of financial and food insecurity, poor access to health insurance and health services or societal discrimination^152^), learning difficulties and social withdrawal. It can be argued that in the case of this particularly vulnerable group of children, it is crucial to address not only their immediate health needs but also their development, future health and well‐being.

#### 4.2.1 Summary

This section has shown that play in many ways contributes to children's healthy development. It strengthens the body, reduces body mass and improves physical health. Play also contributes to children's mental health: it decreases mental distress and helps children regulate their emotions. Moreover, through enhancing cognitive and social skills, play improves children's life prospects and future chances for health. These health‐related benefits refer to all children, but they seem particularly important for vulnerable refugee children, who, in addition to their health disadvantage, experience many barriers to play, which further undermines their well‐being and development. The next section will introduce an additional reason as to why creating opportunities for play is crucial for refugee children's health. Against the background of past and present military conflicts, it will be argued that play is a natural and potent instrument for healing their war‐related trauma.

### 4.3 Therapeutic value of play

Children in Ukraine have a new, popular game, called ‘checkpoint’.^153^ Equipped with toy guns and homemade wooden rifles, they install a makeshift fortification on the roads and stop the passing cars. ‘Glory to Ukraine. Where are you heading?’ They interrogate, before giving the amused driver permission to go further. Sometimes, they collect money for the army, treating it as a sacred duty, a ritual that they carry out with due solemnity. Other times, they play among themselves and let each other pass after reciting a patriotic poem or singing a song. Another popular game is the air raid game. One of the children shouts: ‘Alert! Danger!’ and others run to hiding places. Even more extreme examples of play persevering through the deepest trauma are games played during the Second World War. As Feldman reports, children did not cease play in the ghettos, concentration camps and secret bunkers of the Holocaust.^154^ Probably the darkest one was a game called ‘Going to the Gas Chamber’, in which children of Auschwitz acted out their own death.^155^

These unsettling examples show us the astounding perseverance of play, which lives on even through the most adverse circumstances. As such, they also testify to its vital significance and therapeutic potential. Play constitutes an irreplaceable means to confront, process, and comprehend their traumatic experience of war. It is the natural, universal language of children. Interestingly, the origins of play therapy (not only of war‐related play therapy but also play therapy in and of itself) lie in the experience of World War II. Working with children's refugees at the Hampstead War Nursery, pioneering psychologists Freud and Burlingham observed that whereas only a few children *spoke* about war, war games, especially those involving air raids, were ubiquitous.^156^ Children expressed and communicated their unspeakable experience through play, which thus became recognized as an instrument of therapy (as well as a tool in diagnostics of trauma in children who would not communicate it otherwise). Through play, children organize their memories, integrate fragmented sensory experiences and reconstruct them to increase comprehension.^157^ They put names to the events and feelings, and try to make sense of this experience. As Frankl famously noticed, discovering the meaning of difficult events is a condition for healing and building resilience.^158^ Therefore, if successful, the process of constructing a coherent and meaningful narrative can bring comfort and reassurance to children. Importantly, while playing together, children are not left alone with their troubling feelings and memories. Play gives them an opportunity to share traumatic subjective experience with others and to strengthen attachments and form their own networks of support.^159^

Its mimetic character is what gives play its most therapeutic potential. Play not only mirrors reality and parodies emotions but also represents real events, bringing them in or invoking them through imitative performance.^160^ This was recognized in ancient Greek tragedies, where a theatrical performance was supposed to evoke the experience of catharsis and help citizens of Athens to deal with the adversities or fatalities within their own lives. It was also a familiar element in ancient religious rituals, which invoked the presence of gods through ritualistic gestures, dance and music. In that same way, children can also re‐enter the reality of trauma while playing. Naturally, replaying traumatic events does not come without danger. As Cohenan and Gadassi describe, in negative cases of posttraumatic play, this re‐enactment does not bring soothing; it can instead overwhelm a child with intrusive thoughts and feelings.^161^ This toxic type of play may end up worsening the mental condition of the child if not addressed with professional intervention. However, in most cases, the safe ‘as if’ frames of play give children a chance to transform their traumatic experience of anxiety and fear into feelings of mastery and control. The major healing function of play lies precisely in creating space for this transformation.^162^ For example, by using their make‐believe supernatural capabilities in popular superhero play, children may transform their experience of being a passive victim into an active and empowered individual.^163^ The ubiquitous gun play may help a child to accommodate aggression and regain control over the troubling memories of threat, persecution and helplessness.^164^ In this sense, even a game as horrifying as ‘Going to the Gas Chamber’ may have served the cathartic function of confronting and taming their fear of death.

Play, therefore, is a creative means of healing and self‐protection, which, in most instances, children apply independently (as a ‘self‐guided and self‐healing process’, UNCRC put it). It is important to recognize that the therapeutic value of play lies not only in its professional application but also in the free, unrestrained play of children on their own. Thus, rather than providing actual play activities, a fundamental part of comprehensive play policy should consist of providing opportunities for free play and raising adult awareness of its significance. Heldal gives a compassionate example of an adult accompanying a child in a freely chosen form of play.^165^ While volunteering in a Greek refugee camp, she was invited by a Syrian boy to play a game, described as a ‘plane trip in the tent’. The game consisted of hiding, together with a boy and the toy animals that he gathered, in a tent that was surrounded by a dangerous and hungry tiger. At a certain moment, the boy would ‘start’ a Lego engine and move the whole team to a safe place. As Heldal explains, his freedom in initiating, organizing and entirely managing the content of play—as opposed to being only a passive recipient of the adult's play initiative—was a key element in his regaining control over the tragic events in his life thus far. Additionally, the gentle presence of a chosen adult who respected and followed the child's initiative helped him to restore trust in interactions with others. The presence of a perceptive, trained adult could also benefit the child by recognizing any worrisome or toxic instances of post‐traumatic play and then redirecting the child to professional play therapists. Following the recommendations of UNCRC, the latter should be made an integral part of providing play to the youngest and most vulnerable victims of war.

## 5 CONCLUSIONS AND RECOMMENDATIONS

The Committee on the Rights of the Child rightly observed that in the situation of armed conflict, children's right to play tends to be given a lower priority than other children's needs such as provision of food, shelter and traditional medicines.^166^ Indeed, when children are being killed or wounded and suffering hunger, play seems to be the least of priorities. However, it is precisely in this dramatic situation when children need to play the most. Play can help children explore and find meaning in what has happened to them, regain the sense of control over their lives and recover a sense of joy after traumatic experiences.^167^ Following these findings, this paper has argued for the greater recognition of the right to play for children affected by war trauma. The analysis of UNCRC supported by relevant empirical evidence amounted to the following conclusions and recommendations.

### 5.1 International policy to provide play in refugee camps

Considering that the number of child refugees is reported to be the highest since Second World II and constantly increasing,^168^ there is an urgent need to complete scattered play initiatives with international policy for providing play opportunities to displaced children. While discussing this problem, Collins and Wright^169^ rightly pointed out that, although UNICEF does recognize the importance of play for the healthy development of children and their recovery of trauma of war,^170^ it still lacks systematic programming for children's right to play (for example, neither its actual Strategic Plan 2022–2025 nor its earlier versions included any programming for children's right to play). Consequently, although many international and nongovernmental organizations do provide play opportunities to displaced children, their initiatives are scattered, not sufficiently based on research‐informed standards, lack evaluation and monitoring^171^ and, as was argued on the example of the Children Friendly Spaces, display significant shortcomings in practice.^172^ Therefore, the postulated play policy should include minimum standards for providing play in CFS and guidelines for training staff and volunteers in children‐centred play methodologies, also sensitizing them to difficult subjects that may emerge in posttraumatic play. Moreover, with only a few empirical studies measuring the outcomes of CFSs in emergency areas, there is also a need to strengthen the evidence base regarding their effectiveness, and develop criteria for its evaluation and monitoring.

### 5.2 Play in national policies

The obligation to provide opportunities for play does not end in refugee camps, but should rather follow children into all other places and communities where they may end up. Unfortunately, without international play policy and with very few national strategies for the provision of opportunities to play, little is known about the possible instruments that countries receiving refugees can use to fulfil their obligations under art. 31 UNCRC towards displaced children who live on their territories, and whether they do it in a non‐discriminatory manner—to which they are obliged under art. 2 and art. 22. It is known, however, that, as Irish National Play Strategy (one of the two national strategies adopted with respect to play) reports, refugee children experience several barriers to play, such as a lack of safe spaces, language difficulties, mobility problems, fear of assault, concerns about safety, especially among girls, and an absence of transport.^173^ To respond to this problem, it is recommended that the receiving countries invest more legislative, administrative and financial effort in organizing play infrastructure and creating play opportunities for refugee children living in their cities and towns. This could take the form of a national play policy, including a detailed focus on refugee children or, at a minimum, a set of coordinated play programmes addressing this particular disadvantaged group of children (with initiatives such as pop‐up mobile play^174^ spaces, play activities in parks, schools, community centres or play encounters in cultural institutions such as libraries, theatres or museums). Considering that the UN Committee on the Rights of the Child urged cross‐institutional cooperation in play provision,^175^ different sectors of national, regional and municipal authorities should be engaged in realizing refugee children's rights under art. 31. While providing opportunities to play that specifically address refugee children, it is important to remember that play—as a globally understood language of childhood—is also a natural instrument for their inclusion and integration with local children, and efforts to facilitate such a play‐mediated inclusion should be made.

### 5.3 Play as part of public health strategy

Finally, it has been argued that the obligation to provide play partially falls on the healthcare sector. This thesis, grounded in legal analysis of UNCRC, was supported by the empirical evidence demonstrating how play contributes to children's survival, their general health and recovery from war trauma. In light of this analysis, it is recommended that provision of opportunities to play be included in public health strategies addressing the mental and physical health of refugee and displaced children. The public health approach was not only argued to be the most suitable to ensure the universal enjoyment of the human right to play, but it has also been recommended to respond to increasing and large‐scale needs of the vulnerable population of refugees.^176^ As UNICEF endorsed, providing opportunities for play, along with social support, development and clinical services for specific problems, ‘all constitute important aspects of programming’^177^ for mental health and psychosocial recovery of children victims of war. Following distinctions made in UNCRC, it has been argued that play can be both a natural, self‐guided and self‐healing process of trauma recovery, as well as an instrument in the hand of a play therapist. Accordingly, it is recommended that public health strategy addressing the mental health of refugee children include both the provision of free and child‐centred play (e.g., in schools, community centres and other settings) as well as the organization of play sessions where a professional play specialist or therapist is present. Such play sessions assisted by a play specialist could facilitate the diagnosis of mental health conditions among refugee children and increase access to professional support to those who need it. Considering that parents in a situation of crisis may be inclined to restrict children's opportunity to play,^178^ paediatric health professionals could also be involved as ‘trusted child advocates’^179^ in the campaign to raise awareness among parents, teachers and other child caregivers of the importance of play for the recovery of traumatized children. Moreover, taking into account that many refugee children are physically inactive,^180^ play programmes designed specifically to increase their physical activity (e.g., sport or dance clubs, outdoor activities in city parks, green areas, or car‐free zones) could be an important contribution to the general health of this group of children.

To close with one last word, it is worth noticing that children's play, which proved itself capable of persevering through the darkest times and places, can also be a source of hope and healing for us—adult, concerned observers of the ongoing global events. The ordinary miracle of children's play can lead us to believe that our violence‐stricken world can grow into a healthier and better place.

## Footnotes

1 Finnis, J. (2011). *Natural law and natural rights* (p. 87). Oxford University Press.

2 Nussbaum, M. (2000). *Women and human development: the capabilities approach* (p. 80). Cambridge University Press; Nussbaum, M. (2001). *Upheavals of thought: The intelligence of emotions* (pp. 206–224). Cambridge University Press; Nussbaum, M. (2007). *Frontiers of justice* (p. 77). The Belknap Press of Harvard University Press; Nussbaum, M. (2010). *Not for profit: Why democracy needs the humanities* (pp. 95–120). Princeton University Press; Nussbaum, M. (2011). *Creating capabilities: The human development approach*. Belknap Press of Harvard University Press (pp. 34–36).

3 Nussbaum, M. (1992). Human functioning and social justice: In defense of Aristotelian essentialism, *Political Theory. 20*(2), 219.

4 UN General Assembly. (1989). *Convention on the rights of the child* (vol. 1577). 20 November 1989. United Nations. Treaty Series. https://treaties.un.org/pages/ViewDetails.aspx?src=IND&mtdsg_no=IV-11&chapter=4

5 UNCRC has been ratified by 195 countries, with the only exception of the United States, which signed the Convention on 16 February 1995 but never ratified it. Cf. United Nations Treaty Collection. https://treaties.un.org/pages/ViewDetails.aspx?src=IND&mtdsg_no=IV-11&chapter=4&clang=_en

6 Collins, T., & H. V. Wright, L. (2019). Advancing the right to play in international development. In C. Fenton‐Glynn (Ed.), *Children's rights and sustainable development: Interpreting the UNCRC for future generations* (Treaty Implementation for Sustainable Development, pp. 306–336). Cambridge University Press, p. 319.

7 Hodgkin, R., & Newell, P. (2007). *Implementation handbook for the convention on the rights of the child* (3rd ed., p. 469). UNICEF; Fronczek, V. (2009). Article 31: A 'forgotten article of the UNCRC’. Early child‐hood matters. *Early Childhood Matters, 113*, 24–28.

8 Slone, M., & Mann, S. (2016). Effects of war, terrorism and armed conflict on young children: A systematic review. *Child Psychiatry and Human Development*. *47*(6), 950–965.

9 Van Gils, J. (2007). The child's right to play: the right to be a child. In A. Ang, et al. (Eds.), *The UN children's rights convention: Theory meets practice: Proceedings of the International Interdisciplinary Conference on Children's Rights* (pp. 18‐19). Intersentia.

10 Similar obligations can be derived from article 2 of the UNCRC (principle of nondiscrimination), which, as the Committee on the Rights of the Child explained, obliges State Parties to ensure that all children, in particular asylum‐seeking and refugee children, have the opportunity to realize their rights under article 31 without discrimination of any kind. Cf. UN Committee on the Rights of the Child (UNCRC). (2013). *General comment No. 17 (2013) on the right of the child to rest, leisure, play, recreational activities, cultural life and the arts (art. 31)*. 17 April 2013, CRC/C/GC/17, p. 7.

11 Ibid: 9.

12 Vaghri, Z., Tessier, Z., & Whalen, C. (2019). Refugee and asylum‐seeking children: Interrupted child development and unfulfilled child rights. *Children, 6*(11), 120, p. 1.

13 Kilkelly, U. (2016). The best interests of the child: A gateway to children's rights. In Sutherland, E. E., & Macfarlane, L. A. B. (Eds.). *Implementing article 3 of the United Nations Convention on the rights of the child* (pp. 51–66). Cambridge University Press.

14 Ibid: 75.

15 The concept of well‐being has its roots in the broad WHO notion of health as encompassing social, mental and spiritual dimensions and in the field of social science, which introduces the concept of quality of life. Cf. Ben‐Arieh, A., & Tarshish, N. (2016). Children's Rights and Well‐Being. In M. D. Ruck, M. Peterson‐Badali, & M. Freeman (Eds.), *Handbook of children's rights* (pp. 68–79). Routledge University Press, p. 71.

16 Anderson Moore, K., Lippman, L. H., McIntosh, H. (2009). *Positive indicators of child well‐being: A conceptual framework, measures and methodological issues*. UNICEF: Innocenti Working Papers, no. 2009‐21.

17 Feldman, D. (2019). Children's play in the shadow of war. *American Journal of Play, 11*, 288–307.

18 United Nations. (2022). Ninety per cent of war‐time casualties are civilians, speakers stress, pressing security council to fulfil responsibility, protect innocent people in conflicts. https://press.un.org/en/2022/sc14904.doc.htm

19 Polish News. (2022, May 31). *Azovstal, Mariupol. Anna talks about 65 days underground with her eighteen‐month‐old baby*. https://polishnews.co.uk/azovstal-mariupol-anna-talks-about-65-days-underground-with-her-eighteen-month-old-baby/

20 Hyder, T. (2005). *War, conflict, and play* (p. 24). Open University Press.

21 Ibid: 35–36.

22 Chen, S., & Knöll, M. (2022). Perceived environmental barriers and facilitators of refugee children's physical activity in/around refugee accommodation: A qualitative case study in Berlin. *Archives in Public Health, 80*, 242.

23 UN Secretary‐General (UNSG). (2023). *Children and armed conflict: Report of the Secretary‐General* (pp. 2–3). 27 June 2023. A/77/895‐S/2023/363. https://documents-dds-ny.un.org/doc/UNDOC/GEN/N23/144/96/PDF/N2314496.pdf?OpenElement

24 Narayan, A. J., & Masten, A. S. (2012). Children and adolescents in disaster, war, and terrorism: Pathways to psychopathology and resilience. In C. Widom (Ed.), *Trauma, psychopathology, and violence* (pp. 131–158). Oxford University Press.

25 Bürgin, D., Anagnostopoulos, D., Board and Policy Division of ESCAP, Vitiello, B., Sukale, T., Schmid, M., & Fegert, J. M. (2022). Impact of war and forced displacement on children's mental health‐multilevel, needs‐oriented, and trauma‐informed approaches. *European Child & Adolescent Psychiatry*, *31*(6), 847.

26 For the psychological consequences of this separation, see: Jones‐Mason. K., Behrens, K.Y., Gribneau Bahm, N.I. (2021). The psychobiological consequences of child separation at the border: Lessons from research on attachment and emotion regulation. *Attachment & Human Development*, *23*(1), 1–36.

27 Halevi, G., Djalovski, A., Vengrober, A., & Feldman, R. (2016). Risk and resilience trajectories in war‐exposed children across the first decade of life. *Journal of Child Psychology and Psychiatry, and Allied Disciplines*, *57*(10), 1183–1193.

28 Slone, M., & Mann, S., op. cit. note 5; Blackmore, R., Gray, K. M., Boyle, J. A., Fazel, M., Ranasinha, S., Fitzgerald, G., Misso, M., & Gibson‐Helm, M. (2020). Systematic review and meta‐analysis: The prevalence of mental illness in child and adolescent refugees and asylum seekers. *Journal of the American Academy of Child and Adolescent Psychiatry*. *59*(6), 705–714; Amiri S. (2022). Prevalence of suicide in immigrants/refugees: A systematic review and meta‐analysis. *Archives of Suicide Research: Official Journal of the International Academy for Suicide Research*, *26*(2), 370–405; Joshi, P. T., & O'Donnell, D. A. (2003). Consequences of child exposure to war and terrorism. *Clinical Child and Family Psychology Review*, *6*(4), 275–292.

29 UNICEF. (1993). *Children in war: A guide to the provision of services*. United Nations Children's Fund.

30 Gil, E. (2016). *Posttraumatic play in children: What clinicians need to know*. Guilford Publications.

31 Hyder, op. cit. note 20, p. 49.

32 Cohen, E., & Gadassi, R. (2018). The function of play for coping and therapy with children exposed to disasters and political violence. *Current Psychiatry Reports*, *20*(5), 31–37.

33 Ibid: 32.

34 Landers, C. (1998). *Listen to me: Protecting the development of young children in armed conflict*. Office of Emergency Programs Working Paper Series. UNICEF, p. 12.

35 Winnicott, D. W. (1991). *Playing and reality*. Routledge.

36 Hammer, T.R. (2011). Pretend play. In: Goldstein, S., Naglieri, J. A. (Eds.), *Encyclopedia of child behavior and development* (p. 1157). Springer.

37 Bergen D. (2002). The role of pretend play in children's cognitive development. *Early Childhood Research and Practice*. *4*, 1–13; Smith, L. B., & Jones, S. S. (2011). Symbolic play connects to language through visual object recognition. *Developmental Science*, *14*(5), 1142–1149; Orr, E., & Geva, R. (2015). Symbolic play and language development. *Infant Behavior & Development*, *38*, 147–161.

38 Winnicott, D. W. op. cit, note 35, pp. 104–110.

39 Ogden, T. H. On potential space. In M.B., Spelman & F. Thomson‐Salo (Eds.). (2015). *The Winnicott tradition: Lines of development‐evolution of theory and practice over the decades* (pp. 121–133). Routledge, p. 121.

40 UNCRC. (2013). *General Comment No. 17 (2013) on the right of the child to rest, leisure, play, recreational activities, cultural life and the arts (art. 31)*. 17 April 2013, CRC/C/GC/17.

41 Payà Rico, A., & Bantulà Janot, J. (2021). Children's right to play and its implementation: A comparative, international perspective. *Journal of New Approaches in Educational Research*. *10*(2), 279‐294.

42 IPA. (2010). *Promoting the child's right to play: IPA [International Play Association] global consultations on children's right to play report*. IPA, p. 3.

43 Payà & Bantulà, op. cit. note 41, p. 290.

44 Ibid: 291.

45 Cf. Chatterjee, S. (2017). *Access to play for children in situations of crisis: Synthesis of research in six countries*. IPA.

46 Ager, A., Metzler, J., Vojta, M., & Savage, K. (2013). Child friendly spaces: A systematic review of the current evidence base on outcomes and impact. *Intervention*, *11*(2), 133–147.

47 Ardelean. A. (2021). Play in a refugee camp: disorder from chaos. *International Journal of Play*, *10*(4), 355–360; Heldal, M. (2021). Perspectives on Children's Play in a Refugee Camp. *Interchange, 52*, 433–445; Metzler, J., Savage, K., Yamano, M., and Ager, A. (2015). *Evaluation of child friendly spaces: An inter‐agency series of impact evaluations in humanitarian emergencies*. Columbia University Mailman School of Public Health and World Vision International.

48 Ardelean, A., op. cit. note 47, p. 357.

49 Landers, C., op. cit., note 30, p. 38.

50 Waniganayake, M. (2001). From playing with guns to playing with rice: The challenges of working with refugee children: An Australian perspective, *Childhood Education*, *77*(5), 291.

51 Sims, M., Hayden, J., Palmer, G. and Hutchins, T. (2002). Young children who have experienced refugee or war‐related trauma, *European Early Childhood Education Research Journal*, *10*(1), 106.

52 Ardelean, A., op. cit. note 47, 356.

53 Ibid: 358–359.

54 UNICEF. (2022). *Ukrainian families fear for future as winter bites*. https://www.unicef.org/ukraine/en/stories/ukrainian-families-fear-future-as-winter-bites

55 UNICEF. (2009). *Machel study 10‐year strategic review: Children and conflict in a changing world* (p. 111). https://www.refworld.org/docid/4a389ca92.html

56 Sutton‐Smith, B. (1997). *The ambiguity of play*. Harvard University Press.

57 Cf. Van Gils, J. (2014). Play and well‐being in children's life. In A. Ben‐Arieh, F. Casas, I. Frønes, J. Korbin (Eds.), *Handbook of child well‐being* (pp. 895–910). Springer; Nielsen, L. (2021). Don't downplay “play”: Reasons why health systems should protect childhood play. *The Journal of Medicine and Philosophy*, *46*(5), 586–604; Collins & Wright, op. cit. note 6.

58 Huizinga, J. (1980). *Homo ludens; A study of the play‐element in culture*. Routledge.

59 Wittgenstein, L. (1986). *Philosophical investigations*. G.E.M. Anscombe, & R. Rhees (Eds.). (G.E.M. Anscombe, Trans.). Blackwell (§ 23).

60 Plato. *Laws*. § 803 c‐d.

61 Guardini, R. (1998). *The spirit of liturgy* (pp. 61–73). Herder & Herder Book.

62 Lester, S., & Russell, W. (2010). *Children's right to play: An examination of the importance of play in the lives of children worldwide*. Working Paper No. 57. Bernard van Leer Foundation (p. 7).

63 Spariosu, M. I. (1989). *Dionysus reborn: Play and the aesthetic dimension on modern philosophical and scientific discourse* (pp. 246–258). Cornell University Press; Vaihinger, H. (2021). *The philosophy of ‘as if’* (Routledge Classics). Routledge Taylor & Francis Group.

64 Gray, P. (2013). *Free to learn. Why unleashing the instinct to play will make our children happier, more self‐reliant, and better students for life* (p. 141). Basic Books.

65 Cf. Pellegrini, A.D. (2009). *The role of play in human development*. Oxford University Press.

66 At least since Piaget, J. (1962). *Play, dreams, and imitation in childhood*. Norton; Vygotsky, L. S. (1978). The role of play in development. In M. Cole, V. John‐Steiner, and S. Scribner, E. Souberman (Eds.), *Mind in society development of higher psychological processes* (pp. 92–104). Harvard University Press.

67 Singer, D., Golinkoff, R. M., & Hirsh‐Pasek. K. (2006). *Play=learning: How play motivates and enhances children's cognitive and social‐emotional growth*. Oxford University Press; Pramling Samuelsson I., & Fleer. M. (2009). *Play and learning in early childhood settings: International perspectives*. Springer.

68 Barblett, L. (2010). Why play‐based learning? *Every Child, 16*, 4–5; Pyle, A., DeLuca, C., & Danniels, E. (2017). A scoping review of research on play‐based pedagogies in kindergarten education. *Review of Education*, *5*(3), 311–351; Parker, R., Thomsen, B. S., & Berry, A. (2022) Learning through play at school—A framework for policy and practice. *Frontier in Education*, 7:751801.

69 Payà & Bantulà, op. cit. note 41, p. 283.

70 Howard, J., Jenvey, V., & Hill, C. (2006). Children's categorisation of play and learning based on social context. *Early Child Development and Care, 176*(3/4), 379–393.

71 Ibid. Cf. also Goodhall, N., & Atkinson, C. (2019). How do children distinguish between ‘play’ and ‘work’? Conclusions from the literature. *Early Child Development and Care*, 198, 1695–1708.

72 Howard, J., & McInnes, K. (2013). The impact of children's perception of an activity as play rather than not play on emotional well‐being. *Child: Care, Health and Development*, *39*(5), 737–742.

73 Bogatić, K. (2021). Time for play? Researching the right to play in children's everyday lives with time use diaries. In A. Višnjić‐Jevtić, A. R. Sadownik, & I. Engdahl (Eds.), *Young children in the world and their rights. Thirty years with the United Nations convention on the rights of the child* (pp. 87–101). Springer (p. 90).

74 UNCRC, op. cit. note 40, p. 4.

75 Van Gils, J. (2007). *The UN convention on the rights of the child and the evolution of children's play* (p. 2). ICCP Conference in Brno.

76 UNCRC, op. cit. note 40, p. 5.

77 Ibid: 5–6.

78 Ibid: 6.

79 Goonesekere, S. (2020). The interrelated and interdependent nature of children's rights. In J. Todres, S. M. King, & M. Shani (Eds.), *The Oxford handbook of children's rights law* (pp. 71–98). Oxford University Press.

80 Archard, D. W. (2023). Children's rights. In E. N. Zalta & U. Nodelman (Eds.), (Spring 2023 ed.). The Stanford Encyclopedia of Philosophy. https://plato.stanford.edu/archives/spr2023/entries/rights-children/.

81 Archard, D., & Uniacke, S. (2021). The child's right to a voice. *Res Publica. 27*, 521–536.

82 UNCRC, op. cit. note 40, p. 8.

83 Alen, A., Lanotte, J. V., Verhellen, E., Ang, F., Berghmans, E., & Verheyde, M. (2006). *Commentary on the United Nations convention on the rights of the child, Article 31: The right to leisure, play and culture* (p. 3). Brill.

84 UNCRC, op. cit. note 40, p. 6.

85 Ibid: 4

86 Ibid: 8.

87 As the Committee on the Rights of the Child put it: ‘Early childhood care and development work in many countries focuses exclusively on issues of child survival with no attention paid to the conditions that enable children to thrive’. In UNCRC, op. cit. note 40, p. 13.

88 Collins & Wright, op. cit. note 6, pp. 328–330.

89 Lester & Russell, op. cit. note 62, p. 15.

90 UNCRC, op. cit. note 40, p. 9.

91 Ibid.

92 Ibid.

93 Hodgkin & Newell, op. cit. note 7, p. 472; Shackel, R. (2015). The child's right to play: Laying the building blocks for optimal health and well‐being. In A. B. Smith (Eds.), *Enhancing children's rights connecting research, policy and practice* (pp. 48–610). Palgrave Macmillan; Collins & Wright, op. cit. note 6.

94 Mullen, M. (2014). Getting serious about the human right to play*. Asia Pacific Journal of Sport and Social Science, 3*(2), 130–142,

95 Ibid: 136.

96 UNCRC, op. cit. note 40, p. 10.

97 Ibid: 10.

98 Shackel, op. cit. note 93, p. 52.

99 Shackel, op. cit. note 93, p. 53; Collins & Wright, op. cit. note 6, p. 330.

100 UNCRC, op. cit. note 40, p. 10.

101 Shackel, op. cit. note 8, p. 53.

102 Payà & Bantulà, op. cit. note 41, p. 290.

103 WHO Regional Office for Europe. (2017). *Children's rights in hospital: Rapid assessment checklists* (p. 12). World Health Organization.

104 European Association for Children in Hospital (EACH). (1998). *Charter for children in hospital*. https://each-for-sick-children.org/each-charter/

105 Tonkin A., & Whitaker J. (2019). *Play and playfulness for public health and wellbeing*. Routledge.

106 Tonkin, A. (2019). Playing for healthy brain. In A. Tonkin & J. Whitaker (Eds.), *Play and playfulness for public health and wellbeing* (pp. 20–34). Routledge.

107 As Bayliss and Tonkin put it, ‘The universal nature of play and playfulness makes them potent weapons in the battle against inequality’. Cf. Bayliss, R., & Tonkin, A. (2019). Playful policy. In A. Tonkin & J. Whitaker (Eds.), *Play and playfulness for public health and wellbeing* (pp. 175–190). Routledge (p. 185).

108 Etchells, J. & Tonkin, A. (2019). The playful pursuit of child public health. In A. Tonkin & J. Whitaker (Eds.), *Play and playfulness for public health and wellbeing* (pp. 104–119). Routledge.

109 Milteer, R. M., Ginsburg, K. R., Council on Communications and Media, & Committee on Psychosocial Aspects of Child and Family Health. (2012). The importance of play in promoting healthy child development and maintaining strong parent‐child bond: Focus on children in poverty. *Pediatrics*. *129*(1), e204–e213; Yogman, M., Garner, A., Hutchinson, J., et al; AAP Committee on Psychosocial Aspects of Child and Family Health, AAP Council on Communications and Media. (2018). The power of play: A pediatric role in enhancing development in young children. *Pediatrics, 142*(3), 1–16.

110 Milteer, R. M., op. cit. note 105, pp. 185–186. Cf. also Gray, P. (2011). The decline of play and the rise of psychopathology in children and adolescents. *American Journal of Play*, *3*(4), 443–463.

111 Cohen, E. (2019). Political violence (war and terrorism) and children's play. In P. K. Smith & J. L. Roopnarine (Eds.), *The Cambridge handbook of play: Developmental and disciplinary perspectives* (pp. 649–663). Cambridge University Press.

112 Bürgin, D., et al., op. cit. note 25.

113 The story has also been told in biography of J. Vetulani: Kubisiowska, K. (2022). *Vetulani. Piękny umysł, dzikie serce* (p. 3). Znak.

114 Hodgkin & Newell, op. cit. note 7, p. 469.

115 Finnis, op. cit. note 1.

116 Nussbaum, op. cit. note 2.

117 Graham, K. L., & Burghardt, G. M. (2010). Current perspectives on the biological study of play: signs of progress. *The Quarterly Review of Biology*, *85*(4), 393–418.

118 Ibid: 394.

119 Pellis, S. M., & Pellis, V. C. (2006). Play and the development of social engagement: A comparative perspective. In P. J. Marshall & N. A. Fox. (Eds.), *The development of social engagement: Neurobiological perspectives* (pp. 247–274). Oxford University Press.

120 Spinka, M., Newberry, R. C., & Bekoff, M. (2001). Mammalian play: Training for the unexpected. *The Quarterly Review of Biology*, *76*(2), 141–168.

121 Lester & Russell, op. cit. note 62, pp. 20–22.

122 Bürgin, D., et al., op. cit. note 25, p. 848.

123 Ibid: 849.

124 UNICEF. (2009). *Machel study 10‐year strategic review: Children and conflict in a changing world* (p. 109). UNICEF.

125 Collins & Wright, op. cit. note 6, p. 307.

126 Van Gils, op. cit. note 57, p. 900.

127 Milteer, R. M., et al. op. cit. note 109; Yogman M., et al., op. cit. note 109.

128 Sattelmair, J. R., & Ratey, J. J. (2009). Physically active play and cognition an academic matter. *American Journal of Play, 1*, 365–374.

129 Sutton‐ Smith, op. cit. note 56, p. 198.

130 Nijhof, S. L., Vinkers, C. H., van Geelen, S. M., Duijff, S. N., Marijke Achterberg, E.J., van der Net, J., Veltkamp, R. C., Grootenhuis, M. A., van de Putte, E. M., Hillegers, M. H.J., van der Brug, A. W., Wierenga, C. J., Benders, M. J. N. L., Engels, R. C. M. E., Kors van der Ent, C., Vanderschuren, L. J. M. J., & Lesscher, H. M. B. (2018). Healthy play, better coping: The importance of play for the development of children in health and disease. *Neuroscience and Biobehavioral Reviews*, *95*, 421–429.

131 Isen, A. M. (2008). Some ways in which positive affect influences decision making and problem solving. In M. Lewis, J. M. Haviland‐Jones, & L. F. Barrett (Eds.), *Handbook of emotions* (pp. 548–573). The Guilford Press.

132 Pressman, S. D., Jenkins, B. N., & Moskowitz, J. T. (2019). Positive affect and health: What do we know and where next should we go? *Annual Review of Psychology*, *70*, 627–650.

133 Fredrickson B. L. (2004). The broaden‐and‐build theory of positive emotions. *Philosophical Transactions of the Royal Society of London. Series B, Biological Sciences*, *359*(1449), 1367–1378.

134 Frijda, N. H. (1988). The laws of emotion. *American Psychologist, 43*(5), 349–358. Similarly, Halevi, G., et al., op. cit. note 27.

135 Lester & Russell, op. cit. note 62, p. 25.

136 Sutton*‐*Smith, B. (2003). Vulnerability. In J. L. Roopnarine (Ed.), *Play and educational theory and practice. Play and culture studies* (Vol. 5). Praeger.

137 Ibid: 13.

138 Lester & Russell, op. cit. note 62, p. 25.

139 Hirsh‐Pasek, K., Golinkoff, R. M., Berk L., Singer, D. G. (2009). *A mandate for playful learning in preschool*. Oxford University Press.

140 Resnick M. (2017). *Lifelong kindergarten: Cultivate creativity through projects, passion, peers, and play*. MIT Press.

141 Bedrova, E., &. Leong, D. J. (2003). The importance of being playful. *Educational Leadership*, *60*(7), 50–53.

142 Robinson, C., Treasure, T., O'Connor D., Neylon, D., G. Harrison C., & Wynne, S. (2018). *Learning through play: Creating a play‐based approach within early childhood contexts*. Oxford University Press.

143 Hà, T. A. (2022). Pretend play and early language development—Relationships and impacts: A comprehensive literature review. *Journal of Education*, *202*(1), 122–130.

144 Farné, R. (2005). Pedagogy of play. *Topoi*. *24*, 169–181. Cf. also Gray, op. cit. note 60.

145 Nathan, P., & Pellegrini, A. D. (Eds.) (2010). *The Oxford handbook of the development of play*. Oxford University Press.

146 Bailey, R. (2002). Playing social chess: Children's play and social intelligence. *Early Years, 22*(2), 163–73.

147 Sias, P. M., & Bartoo, H. (2007). Friendship, social support, and health. In L. L'Abate (Ed.), *Lowcost approaches to promote physical and mental health: Theory, research, and practice* (455–472). Springer Science.

148 Halevi, G., et al., op. cit. note 27.

149 Nielsen, op. cit. note 57.

150 Wolff, J., & A. de‐Shalit. (2013). On fertile functionings: A response to Martha Nussbaum. *Journal of Human Development and Capabilities, 14*(1), 161–165.

151 As Nielsen puts it: ‘This is so, because—as I have argued above—children's play functioning has significant instrumental importance for the development of social capabilities and cognitive skills, and therefore the protection of the capability for childhood play will improve the long‐term social determinants of health, and the absence of children's play functioning is likely to have a negative impact on people's future social determinants of health (such as education, unemployment, and social exclusion)’. In Nielsen, op. cit. note 57, p. 599.

152 Feinberg, I., O'Connor, M. H., Owen‐Smith, A., & Dube, S. R. (2021). Public health crisis in the refugee community: Little change in social determinants of health preserve health disparities. *Health Education Research*, *36*(2), 170–177.

153 New York Times. (2022). *The children of war*. https://www.nytimes.com/live/2022/08/06/world/ukraine-russia-war-children

154 Feldman, op. cit. note 17, p. 296.

155 Eisen, G. (1990). *Children and play in the holocaust: Games among the shadows*. University of Massachusetts Press; Opie, I. A., & Opie, P. (1984). *Children's games in street and playground: Chasing catching seeking hunting racing duelling exerting daring guessing acting pretending*. Oxford University Press.

156 Freud A. & Burlingham D. T. (1943). *War and children*. Medical War Books.

157 Cohen & Gadassi, op. cit. note 32, p 32.

158 Frankl, V. (2014). *Man's search for meaning*. Beacon Press.

159 Cf. Collins & Wright, op. cit. note 6, p. 330.

160 Ryall, R., Russell, W., & MacLean, M. (Eds). *The philosophy of play* (p. 3). Routledge.

161 Chazan, S., & Cohen, E. (2010). Adaptive and defensive strategies in post‐traumatic play of young children exposed to violent attacks. *Journal of Child Psychotherapy*, *36*(2), 133–151.

162 Cohen & Gadassi, op. cit. note 32, p. 32.

163 Pynoos, R. S., & Nader, K. (1993). Issues in the treatment of posttraumatic stress in children and adolescents. In J. P. Wilson & B. Raphael (Eds.), *International handbook of traumatic stress syndrome* (pp. 535–549). Plenum Press (p. 536).

164 Hyder, op. cit. note 20, pp. 90–94.

165 Heldal, op. cit. note 47.

166 Cf. UNCRC, op. cit. note 40, p. 17.

167 Ibid: 17.

168 UNICEF. (2022). *Nearly 37 million children displaced worldwide—Highest number ever recorded*. https://www.unicef.org/press-releases/nearly-37-million-children-displaced-worldwide-highest-number-ever-recorded

169 Collins & Wright, op. cit. note 6, p. 321.

170 Cf. UNICEF, op. cit. note 120, pp. 138–141.

171 Ager, A., et al., op. cit. note 46.

172 Ardelean, op. cit. note 47; Heldal, op. cit. note 47.

173 National Children's Office. (2008). *Ready steady play! A national play policy* (p. 28). The Stationery Office.

174 Irish National Play Strategy gives an example of good practice in that respect: To facilitate the integration of vulnerable children into mainstream services, they introduced a Galway Play Bus, whose upper deck was adopted as a play area for the refugee and asylum‐seeking children who did not have equal access to play spaces. In Ibid: 29. Similar recommendations are made in the IPA report: Chatterjee, op. cit. note 45, p. 45.

175 UNCRC, op. cit. note 40, p. 20.

176 Inter‐agency Standing Committee. (2007). *IASC guidelines on mental health and psychosocial support in emergency settings*. Geneva: IASC. WHO. (2018). *Report on the health of refugees and migrants in the WHO European Region. No public health without refugee and migrant health*. Copenhagen: WHO; WHO Regional Office of Europe. (2022). *Ukraine crisis. Public Health Situation Analysis: Refugee‐hosting countries*. Copenhagen: WHO.

177 UNICEF, op. cit. note 124, p. 141.

178 Chatterjee, op. cit. note 45, p. 34.

179 Milteer, R. M. et al. op. cit. note 109, p. 184.

180 Chen & Knöll, op. cit. note 22, p. 2.

